# Evaluation of occupational safety of pre-hospital emergency personnel in traffic accidents: a qualitative study in Isfahan City

**Published:** 2019-08

**Authors:** Reihaneh Rastegari, Alireza Kaikhai, Morteza Ghaderi

**Affiliations:** ^*a*^ Emergency Medical Service, Isfahan University of Medical Sciences, Isfahan, Iran.

## Abstract

**Background:**

Traffic incidents are the first service provided by pre-hospital emergency staff. The existence of occupational safety for this cortex increases the efficiency and quality of service. This research was conducted to evaluate the occupational safety of pre-hospital emergency personnel in Isfahan by examining their experiences.

**Methods:**

This study was a qualitative study and its purposeful sampling. The degree of entry of individuals into the study of work experience over 3 years as a technician in urban and road bases in the emergency medical section. In this research, 15 people were interviewed and the framework analysis was used to analyze the data.

**Results:**

The findings of the occupational safety assessment study of the pre-hospital emergency staff with their experiences of four main axes and 16 sub-axes. The four main axes of personnel experience in occupational safety included: individual problems, organizational problems, inadequate coordination and community-related problems. Personnel 115 considered major issues and problems that led to an increase in job insecurity in people s lack of awareness of 115 emergency tasks. Relief from 115 personnel in cases of assault, psychiatric disorders and anger in the patient s companions, in turn, compromise the safety of 115 personnel. The failure of equipment and vehicles and the lack of recognition of some natural and chemical and microbial incidents before deployment lead to increased incidents and deaths for personnel.

**Conclusions:**

According to the findings of the study, the preparation of a joint message center between the relief agencies, the psychotherapy team and the rapid reaction teams for the relief of particular cases, the coordinated system of payment of salaries, the creation of job rotation between urban and road bases, in Considering the amenities and the non-material needs of the constructive suggestions for improving the personnel performance and improving the job safety of the city of Isfahan.

**Keywords:**

Occupational safety, Emergency, Traffic accidents

